# Characterizing a Safe Approach to Timing of Elective Abdominal Surgery Following Traumatic Pneumothorax: A Case Report and Review of the Literature

**DOI:** 10.1002/ccr3.71215

**Published:** 2025-10-14

**Authors:** Khang Duy Ricky Le

**Affiliations:** ^1^ Department of General Surgical Specialties The Royal Melbourne Hospital Melbourne Victoria Australia; ^2^ Department of General Surgery Northeast Health Wangaratta Wangaratta Victoria Australia; ^3^ School of Medicine, Faculty of Health Deakin University Geelong Victoria Australia; ^4^ Department of Medical Education, Faculty of Medicine, Dentistry and Health Sciences The University of Melbourne Melbourne Victoria Australia

**Keywords:** abdominal surgery, laparoscopic surgery, perioperative optimisation, pneumothorax, trauma, traumatic pneumothorax

## Abstract

A traumatic pneumothorax is a potentially life‐threatening injury that can occur following chest trauma. For large pneumothoraces, the standard of management is decompression with intercostal catheters to avoid significant outcomes such as tension pneumothorax and obstructive shock. This is well described within trauma guidelines; however, there remains a lack of consensus about the appropriate time to undergo safe surgery following the resolution of a traumatic pneumothorax. The approach to decompression involves careful considerations in balancing risks and benefits to the patient, namely that of re‐accumulation of a pneumothorax with positive‐pressure ventilation balanced with the benefit of undergoing surgery. Herein, we report a case that highlights key decision‐making surrounding the time to safe surgery following the resolution of a traumatic pneumothorax for a patient with a new diagnosis of a malignant renal lesion. Our case highlights two key messages: (1) Safe elective abdominal surgery can be performed after 4 weeks from resolution of a traumatic pneumothorax and (2) Multidisciplinary collaboration of perioperative and operative clinicians, including forward planning with the presence of chest decompression equipment at the time of surgery, is highly important to allow efficient and timely management of potential reoccurrence of a pneumothorax.

**Abbreviations**
CPAP: continuous positive airway pressure
CT: computed tomography
CXR: chest x‐ray
ETCO_2_
: end tidal carbon dioxide
ETO_2_
: end tidal oxygen
g: grams
ICU: intensive care unit
L: liters
mL: milliliters
WHO: World Health Organization
μg: microgram
μmol: micromol

**Summary**

- Safe elective abdominal surgery can be performed after 4 weeks from resolution of a traumatic pneumothorax.
- Multidisciplinary collaboration of perioperative and operative clinicians including forward planning with the presence of chest decompression equipment at the time of surgery is highly important to allow efficient and timely management of potential reoccurrence of a pneumothorax.

## 1 Background

Traumatic pneumothoraces are an important sequela of thoracic trauma, accounting for an estimated 40%–50% of chest trauma‐related injuries [1]. Early recognition and management of the patient with a traumatic pneumothorax is paramount. Importantly, although the majority of traumatic chest injuries can be managed conservatively, complications associated with a large traumatic pneumothorax include tension pneumothorax and obstructive shock [2]. Chest decompression with intercostal catheters is indicated for these situations to prevent these complications [2, 3]. These management strategies are well established in trauma guidelines and have led to significant improvement in outcomes for the patient with chest trauma.

There remains a lack of consensus surrounding the risk of re‐accumulation with or without progression to tension pneumothorax for patients exposed to varying degrees of atmospheric pressure or pressure‐related stress to the lungs. This is particularly relevant for clinicians who provide advice about commercial flying for the patient who has recently recovered from a pneumothorax. The theoretical risk is that with reduction in cabin pressure at altitude, Boyle's law dictates expansion in gas volume which could lead to re‐accumulation, tension pneumothorax, and death [4]. Many medical associations provide an arbitrary waiting period of 2 weeks prior to flying; however, these guidelines lack robust scientific evidence to support these recommendations [5, 6, 7, 8]. These arguments also extend to elective surgery, where the risk of developing a pneumothorax may occur in the setting of positive‐pressure ventilation or barotrauma [9, 10]. Currently, there is no current evidence‐informed guideline suggesting when it is safe for patients to undergo general anesthesia, intubation, ventilation, and abdominal surgery in the elective setting. This case report describes a safe multi‐disciplinary approach to elective abdominal surgery with a minimum wait of 4 weeks for a patient with a traumatic pneumothorax.

## 2 Case Presentation

The patient provided written consent for the de‐identification and use of their medical information and data for the generation and publication of this case report.

### 2.1 History and Examination

A 64‐year‐old functionally well man presented with 4 days of acute dyspnoea 1 week after striking the left side of his chest wall on a stainless‐steel fridge door. Past history was significant for depression, benign prostatic hyperplasia, type 2 diabetes mellitus, obstructive sleep apnoea on continuous positive airway pressure (CPAP) therapy, and a prior laparoscopic cholecystectomy. Initial examination was significant for tachypnoea with a respiratory rate of 26 breaths per minute, oxygen saturation of 90% on room air, which improved to 100% with 15 L oxygen applied via a non‐rebreather mask, midline trachea, and reduced breath sounds on the left hemithorax. His heart rate and blood pressure were within normal limits.

### 2.2 Differential Diagnosis, Investigations, and Management

**FIGURE 1:**
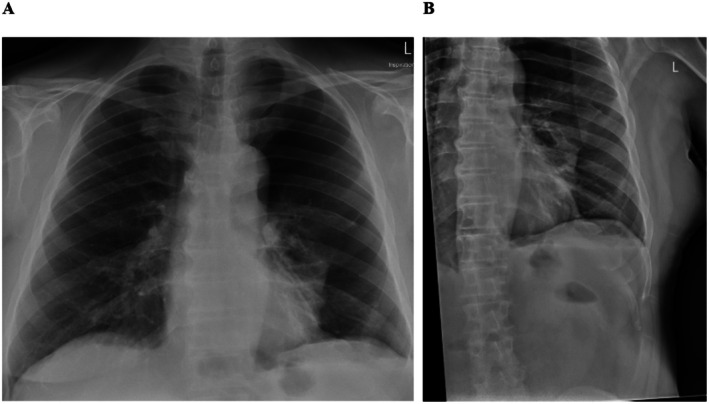
(A) Chest x‐ray in anteroposterior view demonstrating large left sided pneumothorax. (B) Chest x‐ray with focus on the left hemithorax to accentuate the presence of a large left sided pneumothorax.

His initial biochemistry was unremarkable, with a hemoglobin of 141 g/L, white cell count of 4.9 × 10^9^/L, platelets of 201 × 10^9^/L, creatinine of 65 μmol/L, and c‐reactive protein < 1 mg/L. Given concerns about delayed trauma and the oxygen requirement of the patient, a chest x‐ray (CXR) was performed, which revealed a large left pneumothorax with no chest wall bony injuries identified (Figure 1A,B).

**FIGURE 2:**
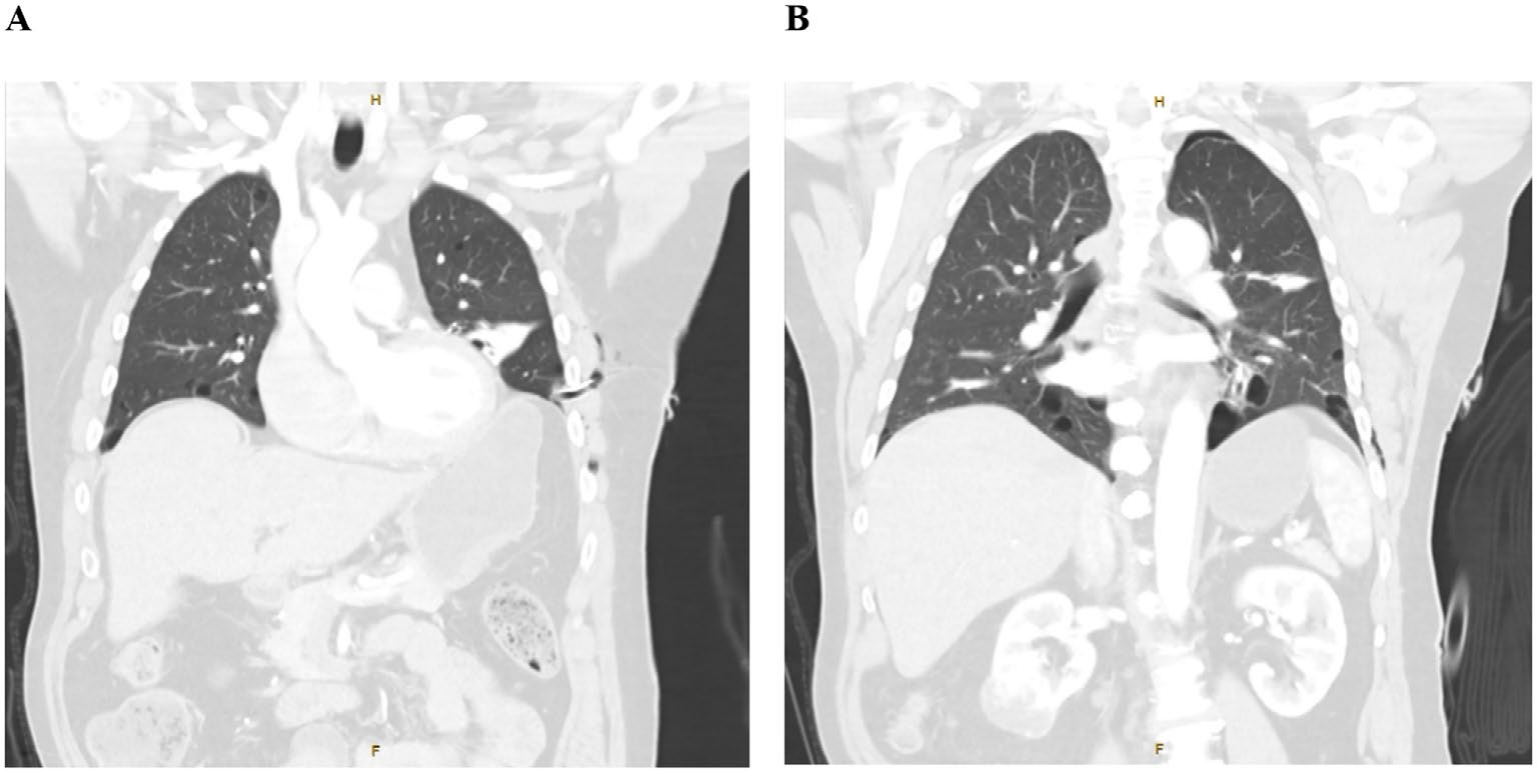
(A) Computed tomography scan of the chest in the coronal plane indicating the presence of an appropriately sited pigtail intercostal catheter entering the left pleural cavity. (B) Computed tomography scan of the chest in the coronal plane indicating the presence of a small residual apical pneumothorax following the insertion of a pigtail catheter.

**FIGURE 3:**
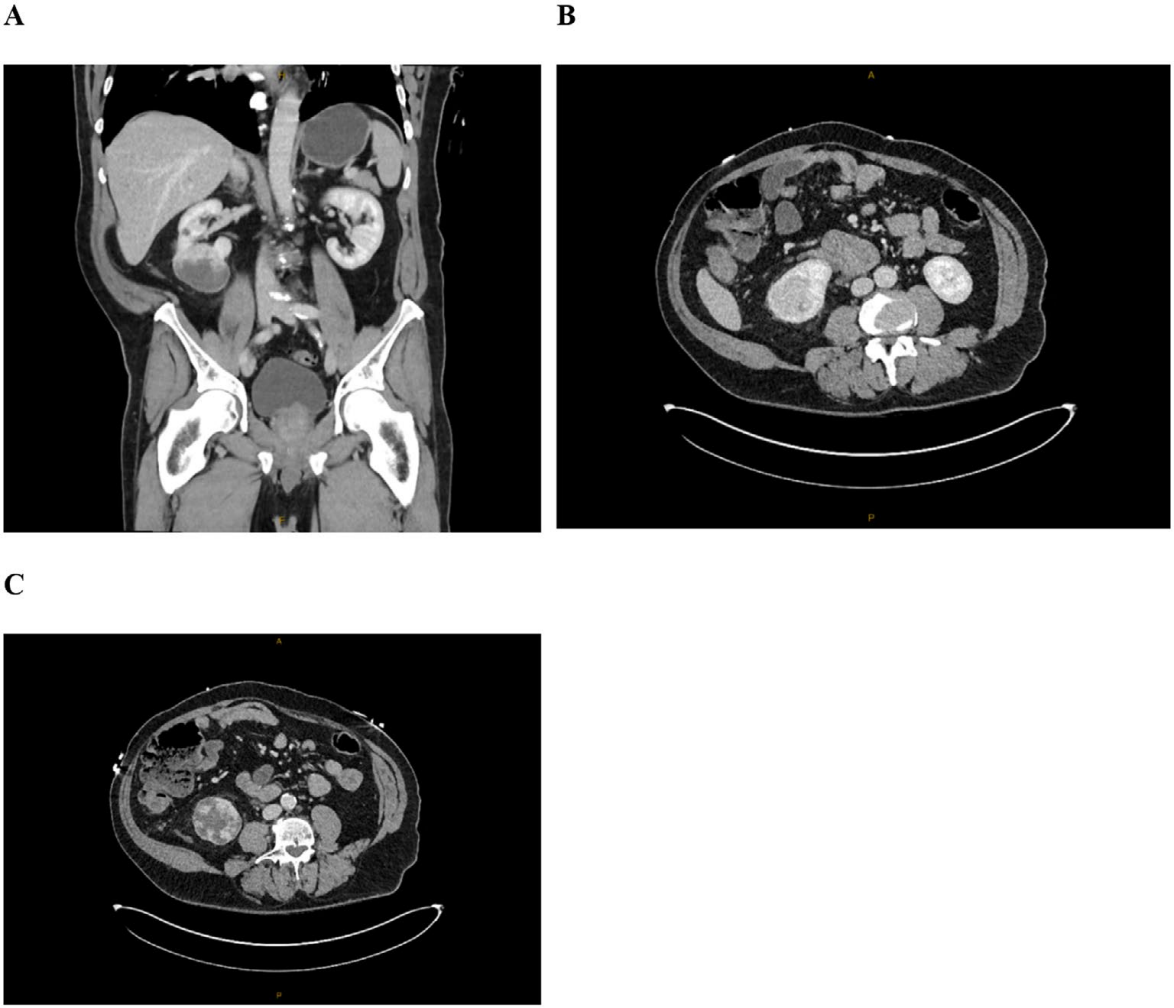
(A) Computed tomography scan of the abdomen and pelvis in the coronal plane indicating a 65 mm heterogeneous vascular mass that was identified arising from the lower pole of the right kidney. (B, C) Computed tomography scan of the abdomen and pelvis in the axial plane indicating a 65 mm heterogeneous vascular mass that was identified arising from the lower pole of the right kidney.

A pigtail intercostal catheter was inserted for immediate decompression of the left‐sided pneumothorax, followed by completion of trauma imaging with a computed tomography (CT) scan of the chest, abdomen, and pelvis, which revealed no further injuries and a residual basal pneumothorax following placement of the pigtail catheter (Figure 2A,B). Incidentally, a 65 mm heterogeneous vascular mass was identified arising from the lower pole of the right kidney, suspicious for renal cell carcinoma (Figure 3).

The patient was observed routinely with daily CXR and clinical assessment with improved oxygenation and no air leak observed from the pigtail catheter. The latter was removed on the third day of admission. Given the renal mass, the patient was planned for laparoscopic right radical nephrectomy, which was complicated by decision making regarding the optimal time to perform safe surgery in the context of recent traumatic pneumothorax. The consensus from the surgical and anesthetic team was that a 4‐week delay to oncological surgery would be appropriate to ensure the risk of re‐accumulation intra‐operatively was minimized. The patient was discharged with a plan for elective surgery after 28 days.

### 2.3 Outcome and Follow‐Up

The patient proceeded to laparoscopic radical right nephrectomy 40 days following discharge. A pre‐operative briefing was held which included a plan for arterial monitoring, judicious airway and ventilation monitoring, and having a chest drain set on standby in the case of re‐accumulation with or without tension of the prior traumatic pneumothorax. These steps were re‐discussed during the performance of the World Health Organization (WHO) safer surgery time out procedure. After pre‐oxygenation, the patient was anesthetized following a total intravenous anesthesia approach using fentanyl 250 μg, rocuronium 100 mg, and propofol 180 mg. Intubation was performed routinely with a size 4 mac blade and a size 8 endotracheal tube. The patient remained stable with mechanical ventilation, end tidal carbon dioxide (ETCO_2_) ranging from 28/0 to 51/0 and end tidal oxygen (ETO_2_) ranging from 45/53 to 65/82. His heart rate and blood pressure remained within normal limits. Pneumoperitoneum was maintained with a pressure of 15 mmHg with no complications, and the nephrectomy was performed in a routine manner. The patient was monitored uneventfully in the intensive care unit (ICU) and stepped down to the ward 2 days after. He was discharged on the third day of admission without any issues.

## 3 Discussion

There remains a lack of consensus surrounding the safe and appropriate timing of elective surgery following the resolution of a traumatic pneumothorax. Similarities to this are drawn from advice about flying after the resolution of pneumothoraces, where patients face variations in pressure that occur in the lungs due to altitude. Current guidelines for flying suggest a 2‐week waiting period and consider an active pneumothorax to be an ‘absolute contraindication’ to flying due to the risk of re‐accumulation and tension [6]. However, conflicts in recommendations also exist, with experts recommending only a 1‐week waiting period following radiographic resolution of a pneumothorax and 2–3 weeks if thoracic procedures have been performed [11]. Further discrepancies in advice, however, are noted within the thoracic surgery community, with highly variable waiting periods of 42 days (mean 13.8 days ± standard deviation of 11.6 days) proposed [11]. Evidently, despite the advancements in evidence‐based thoracic trauma guidelines, there remains significant heterogeneity and ambiguity in recommendations for safe travel following resolved pneumothoraces.

For clinicians, these management dilemmas related to decision‐making for safe surgery following resolution of a traumatic pneumothorax can have a significant impact on the trajectory and experience of care for patients. In particular, our case highlights the new finding of a renal lesion highly suspicious for malignancy. Understandably, such news for a patient can be highly distressing, particularly in the context of delaying treatment. Furthermore, from an oncological perspective, there is a time window in which operative intervention is necessary to allow for curative intent resection and to avoid disease progression. Despite this, there is a need to balance the risk of disease progression with that of immediate harm that may result from expedited surgery. In particular, there are reports of pneumothorax development following endotracheal intubation and laparoscopic surgery [12, 13]. Furthermore, the identification of a pneumothorax in the anesthetized patient is more complex, requiring consideration of parameters such as the peak inspiratory airway pressure, ETCO_2_, and insufflation pressures. In the context of surgery, there may be delays in recognizing minor changes in these parameters due to a focus on other operative priorities.

The lack of consensus about time to safe surgery following resolution of a pneumothorax leads to difficult decision‐making that requires a multidisciplinary approach to navigating, as demonstrated in our case. Key aspects of consideration in the surgical approach to our case include a thorough pre‐operative assessment, preparation of necessary equipment for urgent management including the presence of a chest tube trolley within the operating theater, clear operative plan communication in the pre‐operative phase as part of the WHO time‐out procedure, and judicious monitoring of ventilation parameters during the case. Furthermore, with respect to timing to safe surgery, a consensus was achieved following multidisciplinary collaboration that took into account various guidelines related to flying as well as the preference of local anesthetists. Our case report therefore highlights that following evidence‐based management of a traumatic pneumothorax with decompression with serial monitoring, safe elective abdominal surgery is possible through a well‐planned multidisciplinary approach with a minimum wait of 4 weeks following pneumothorax resolution.

## Author Contributions

**Khang Duy Ricky Le:** conceptualization, data curation, formal analysis, investigation, methodology, project administration, resources, validation, visualization, writing – original draft, writing – review and editing.

## Disclosure

The author has nothing to report.

## Ethics Statement

The case report generation process was discussed with our local ethics and governance team. No formal ethics approval was required following the discussions and therefore was waived.

## Consent

The patient provided informed consent that was written and signed for the generation and publication of this manuscript using their de‐identified medical information.

## Conflicts of Interest

The author declares no conflicts of interest.

## Data Availability

Data can be requested from the corresponding author when required. All relevant data have been provided in the generation of this manuscript, which is intended for open access publication.
